# Unique behavioural modifications in the web structure of the cave orb spider *Meta menardi* (Araneae, Tetragnathidae)

**DOI:** 10.1038/s41598-020-79868-w

**Published:** 2021-01-08

**Authors:** Daniel Simonsen, Thomas Hesselberg

**Affiliations:** Department of Zoology, University of Oxford, Oxford, OX1 2PS UK

**Keywords:** Behavioural ecology, Animal behaviour, Entomology

## Abstract

In the last decade there has been a renewed interest in the study of behavioural adaptations to environmental constraints with a focus on adaptations to challenging habitats due to their reduced ecological complexity. However, behavioural studies on organisms adapted to nutrient poor subterranean habitats are few and far between. Here, we compared both morphological traits, in terms of relative leg lengths, and behavioural traits, captured in the geometry of the spider web, between the cave-dwelling spider, *Meta menardi*, and two aboveground species from the same family (Tetragnathidae); *Metellina mengei* and *Tetragnatha montana*. We found that the webs of the cave spider differed significantly from the two surface-dwelling species. The most dramatic difference was the lack of frame threads with the radii in the webs instead attaching directly to the surrounding rock, but other differences in relative web size, web asymmetry and number of capture spiral threads were also found. We argue that these modifications are likely to be adaptations to allow for a novel foraging behaviour to additionally capture walking prey within the vicinity of the web. We found only limited evidence for morphological adaptations and suggest that the cave orb spider could act as a model organism for studies of behaviour in energy-poor environments.

## Introduction

The study of behavioural adaptations in response to environmental drivers is impeded by a number of factors including our limited knowledge of the phylogenetic relationships of most organisms and hence possible evolutionary constraints, the restrictions arising from the ghost of selection past in many dynamic environments that limits the potential for further selection and the very complex biotic and abiotic interactions present in most ecosystems^1^. One fruitful approach for overcoming some, or most, of these impediments is to investigate novel behaviours in resource-limited environments, which are usually more stable, less species rich and less complex than more benign ecosystems.

Caves and other subterranean habitats constitute a particularly promising ecosystem for in depth studies of behavioural adaptations. The set of common abiotic and biotic environmental characteristics shared by the majority of subterranean habitats influence almost every aspect of an organism’s nature. These common characteristics include absence of light and the consequently nutrient poor conditions and reduced environmental variation, which all add up to produce a stable yet challenging environment^2^. These unique conditions have resulted in the evolution of convergent, morphological adaptations such as the elongation of limbs and the loss of eyes and pigmentation^3^. Similarly, the ecological significance of behavioural traits can be readily explored as the low functional biodiversity, simple trophic webs and limited resources^4^, make it easier to link a trait to the organism’s interactions with the physical or biological environment than in more complex systems^5^. However, the study of behavioural traits in, and their adaptation to, subterranean habitats remains poorly explored with only a few well documented examples^6^, including lack of territorial and aggressive behaviours in both cave amphibians^7,8^ and cave fishes^9,10^, a switch from abdominal vibrations (which transmit poorly in rock) to the use of antenna during courtship behaviour in cave crickets^11^ and the evolution of a novel sit-and-wait foraging strategy through active luring with bioluminescence in glow worms^12^. The paucity of studies is probably mainly due to the non-trivial problems associated with conducting behavioural studies in these inhospitable surroundings and the issues in replicating natural conditions and behaviours in the laboratory^13^.

Spiders are some of the most dominant predators in cave ecosystems with more than a 1000 species exclusively found underground with an equal, if not larger, number of species spending part of their lifecycle in subterranean habitats^14^. A large proportion of these species are web-building sit-and-wait hunters; more than two thirds of recorded cave spiders found in European caves fit into this category, and surprisingly, given the scarcity of flying prey, at least 9 species in the orb weaving family Tetragnathidae inhabit caves^15^.

Orb spiders are excellent model organisms for studying behavioural adaptations as their highly structured two-dimensional webs constitute a direct record of their web building behaviour, and thus also foraging behaviour, that can be easily quantified through measurements of a multitude of geometric variables from photographs^16,17^. The standard orb web consists of a hub, where the spider typically resides, placed slightly above the geometric centre of the oval shaped web from which radii radiate outwards like spokes on a wheel. A sticky capture spiral overlays the radii with the entire structure enclosed within a frame, from which a number of mooring threads connect the web to the surrounding vegetation^18^. This structure is remarkably conserved within the two major orb spider families (Araneidae and Tetragnathidae) with only occasional minor modifications^19–21^, although exceptionally a few species show a complete elimination of webs including adult araneid bolas spiders^22^, and in some adult tetragnathid spiders in the genus *Pachygnatha*^23^ and in the Hawaiian ‘spiny-leg’ clade of *Tetragnatha*^24^. What makes orb spiders particularly interesting model organisms for behavioural studies is that, despite this conservation of the overall web-structure, they show a remarkable flexibility in fine tuning their webs to a large range of biotic and abiotic variables^25–27^. In addition, the evolution of the orb webs and the phylogenetic relationships between the different families and genera of orb spiders are relatively well understood^28,29^.

Cave orb spiders in the genus *Meta* have been anecdotally reported to show behavioural adaptations to nutrient poor subterranean habitats by modifying their webs, including eliminating the frame and reducing overall silk investment, and by potentially engaging in off-web prey capture^15,30–32^. In this study, we compare the web geometry of the European cave orb spider *Meta menardi* with two surface-dwelling tetragnathids; the closely related *Metellina mengei* and the more distantly related *Tetragnatha montana*^33^. As orb spiders generally have poor vision, construct their webs at night and can build normal webs in complete darkness in the laboratory^18,34^, we do not expect the darkness in the caves to affect web geometry per se. However, given the sensitivity of orb webs to fluctuations in temperature, humidity, wind speed and prey type^25,35^, we hypothesise that the stable conditions inside caves, and the rarity of suitable flying prey, will result in significant differences between the webs of cave and aboveground spiders.

During web-building, orb spiders measure distances between spiral loops with their legs^36^ and leg length affects the distances between spiral loops^37^. We therefore also compared leg length and general morphology between our three species to control for any effect of this on web geometry. Since cave adapted animals usually have elongated limbs^3^ our expectation was that *Meta menardi* has longer relative leg lengths than the two aboveground species.

## Results

We encountered a total of 19 spiders and webs of *Meta menardi* from caves near Nottingham, U.K, a total of 29 *Metellina mengei* from woods near Oxford, U.K. and a total of 25 *Tetragnatha montana* near rivers in Oxford, U. K.

### Size and leg length

**Figure 1:**
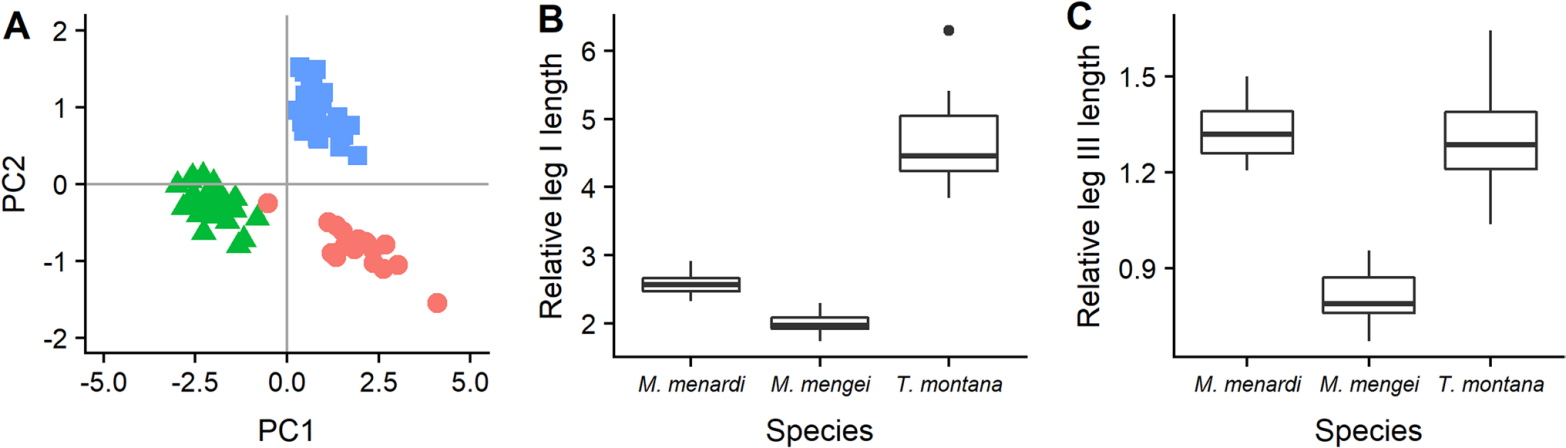
Morphological measurements. (**A**) A principal component ordinal plot of the morphological parameters (total length, cephalothorax width, patella-tibia length of leg I and leg III) of *Meta menardi* (red circles), *Metellina mengei* (green triangles) and *Tetragnatha montana* (blue squares). The combined proportion of variance explained by PCA1 and PCA2 was 98%. (**B**) The relative patella-tibia length (length divided by cephalothorax width) of leg I. (**C**) The relative patella-tibia length (length divided by cephalothorax width) of leg III. The figure was created using the *ggplot2* package^78^ in R^81^.

In terms of overall size, all three species differed from each other as is evident from the principal component analysis (PCA) ordination plot (Fig. 1A). The cave orb spider, even though the spiders measured in this study were late instar juveniles, was larger than the adults of two non-cave tetragnathid species in terms of cephalothorax width (mean ± SEM, *M. menardi*: 2.57 mm ± 0.10 vs. *M. mengei*: 1.56 mm ± 0.03 and *T. montana*: 1.72 mm ± 0.04), but was similar in total length to *T. montana* (8.28 mm ± 0.32 vs. 8.81 mm ± 0.16) with both being longer than *M. mengei* (4.47 mm ± 0.10). However, when we control for size by looking at relative leg lengths (total leg length divided by cephalothorax width), the cave orb spider does not stand out compared to the aboveground species (Fig. 1). While there was a significant difference in the relative lengths of both leg I (Linear mixed model (LMM): F = 310.3, df = 2, *P* < 0.0001) and leg III (LMM: F = 162.1, df = 2, *P* < 0.0001), the outstanding species for either measure was a non-cave one (*T. montana* for leg I and *M. mengei* for leg III). A posthoc test showed that the cave spider had longer relative leg lengths than *M. mengei* for both leg I (t = 6.16, df = 10.25, *P* = 0.0003) and leg III (t = 14.73, df = 6.42, *P* < 0.0001). Significant differences were found also between *M. menardi* and *T. montana* for leg I (t = − 13.51, df = 9.46, *P* < 0.0001), but not leg III (t = 0.91, df = 5.18, *P* = 0.659), and between *M. mengei* and *T. montana* for leg I (t = − 24.84, df = 12.64, *P* < 0.0001) and leg III (t = − 15.293, df = 10.93, *P* < 0.001)(Fig. 1).

### Orb web geometry

**Figure 2:**
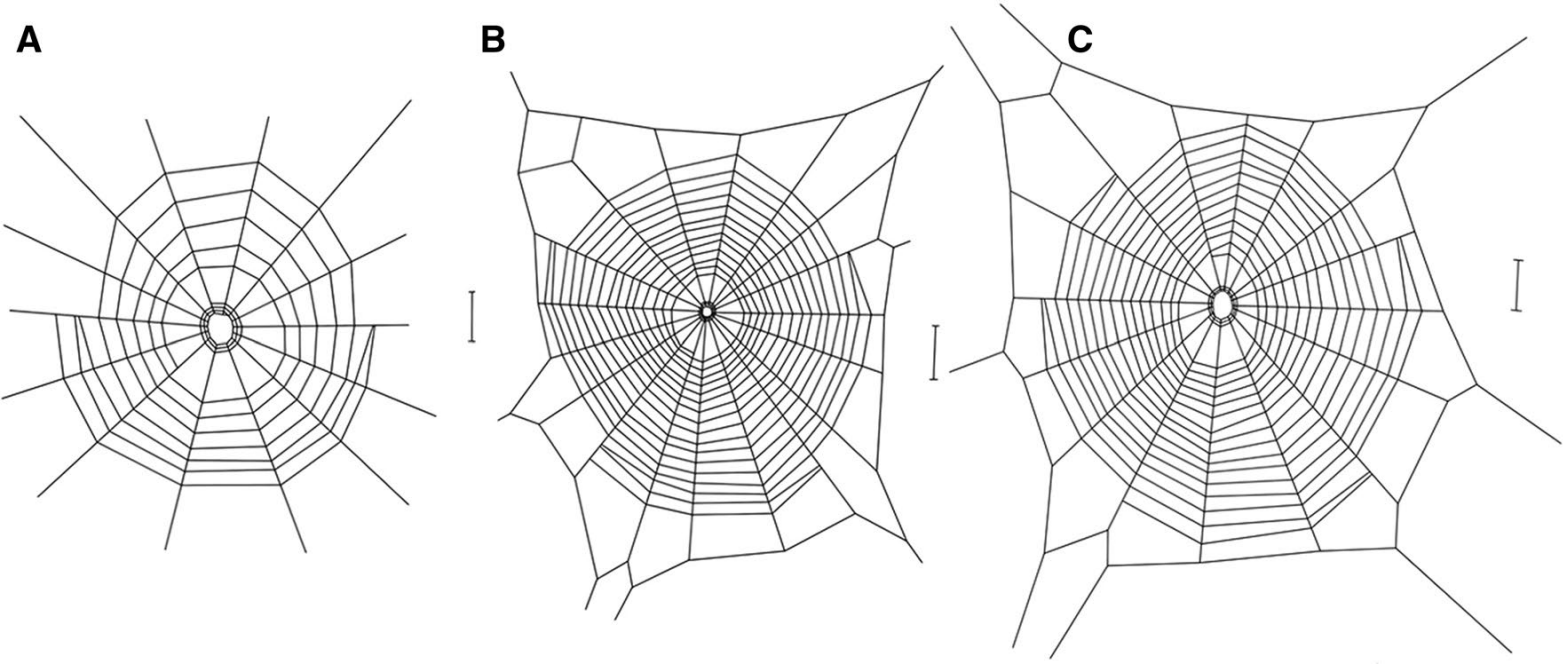
Orb webs of the three species represented as schematic drawings based on detailed observations of their webs in nature. (**A**) *Meta menardi*. (**B**) *Metellina mengei*. (**C**) *Tetragnatha montana*. The vertical scale bar on the right represents 20 mm in length.

**Figure 3:**
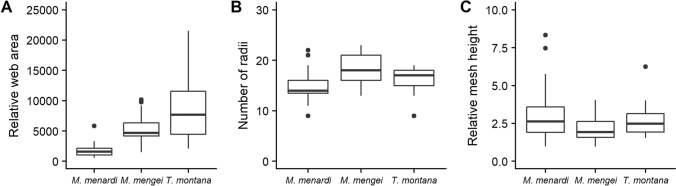
Boxplots of web characteristics of *Meta menardi* (N = 19), *Metellina mengei* (N = 29) and *Tetragnatha montana* (N = 25). (**A**) Relative web area (capture spiral area divided by cephalothorax width squared). (**B**) Number of radii in the web. (**C**) Relative mean mesh height (average mesh height across all four cardinal directions divided by the cephalothorax width of the spider). The figure was created using the *ggplot2* package^78^ in R^81^.

**Figure 4:**
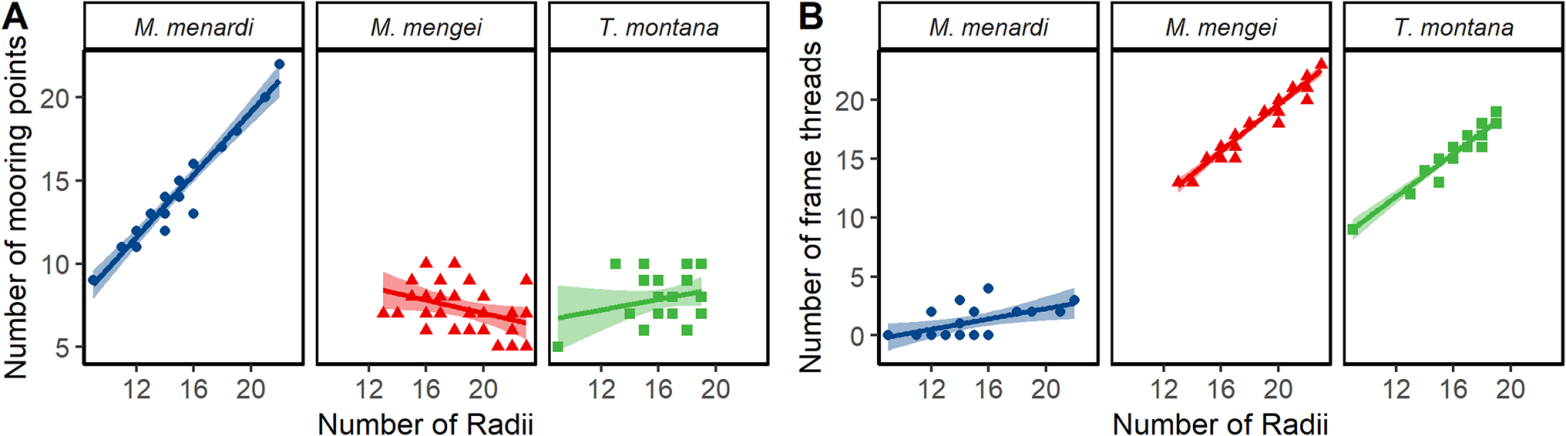
Linear relationship between the number of radii and the number of mooring points (**A**) and the number of frame threads (**B**) in the webs of the cave spider *Meta menardi* (first panel), the forest-dwelling spider *Metellina mengei* (second panel) and the river dwelling *Tetragnatha montana* (third panel). The line indicates the standard regression line with the 95% confidence interval. The figure was created using the *ggplot2* package^78^ in R^81^.

A visual comparison of the overall structure of webs of the three species, strongly indicates a difference between them. The webs of the cave spider *M. menardi* were smaller with fewer spirals and most noticeably had few or no frame threads (Fig. 2). When analysing the web characteristics individually, significant differences were found for relative web area (total web area divided by cephalothorax width squared), where *M. menardi* webs were smaller than the webs of the two aboveground species (Fig. 3A, LMM: F = 39.8, df = 2, *P* = 0.0004;* M. menardi*–*M. mengei* posthoc: t = − 6.95, df = 5.59, *P* = 0.0014; *M. menardi–T. montana* posthoc: t = − 8.69, df = 4.12, *P* = 0.0019), while *M. mengei* and *T. montana* had similar sized webs (t = − 2.39, df = 10. 25, *P* = 0.087). However, only non-significant differences were found between the species for the number of radii (Fig. 3B, LMM: F = 3.26, df = 2, *P* = 0.076) and relative mesh height (total mesh hight divided by cephalothorax width) (Fig. 3C, LMM: F = 4.37, df = 2, *P* = 0.068). As stated, the most obvious visual difference between the webs is the lack of frame threads with the radii attaching directly to the substrate resulting in the radii, in effect, acting as mooring threads in the cave spider *M. menardi*. This dramatic difference is further highlighted when visualizing the linear relationship between the number of radii and mooring threads (Fig. 4A) and number of frame threads (Fig. 4B). Unsurprisingly, the statistical analysis supports this as *M. menardi* had significantly more mooring threads (LMM: F = 23.5, df = 2, *P* < 0.0001; *M. menardi–M. mengei* posthoc: t = 6.73, df = 13.0, *P* < 0.0001; *M. menardi–T. montana* posthoc: t = 5.64, df = 12.3, *P* = 0.0003) and significantly fewer frame threads (LMM: F = 118.8, df = 2, *P* < 0.001; *M. menardi–M. mengei* posthoc: t = − 15.25, df = 10.32, *P* < 0.0001; *M. menardi–T. montana* posthoc: t = − 12.05, df = 9.54, *P* < 0.0001), while the aboveground species showed no differences in the number of mooring threads (t = − 0.74, df = 13.5, *P* = 0.751), but a marginal difference in the number of frame threads (t = 2.77, df = 12.66, *P* = 0.041)(Fig. 4). Significant differences between the species were also found in vertical web asymmetry, absolute web area and number of capture spiral turns, but not in web shape (Table 1). The cave spider *M. menardi* had the most symmetric web, although it did not differ significantly from the webs of either *M. mengei* (t = 2.85, df = 5.59, *P* = 0.070), or *T. montana* (t = 3.03, df = 4.12, *P* = 0.079). Similarly, *M. menardi* webs had the least number of spiral turns, which differed significantly from both the webs of *M. mengei* (t = − 7.56, df = 7.77, *P* = 0.0002) and *T. montana* (t = − 6.49, df = 6.66, *P* = 0.0010), while the latter two had a similar number of spiral turns (t = 0.75, df = 11.64, *P* = 0.739). *Tetragnatha montana* had the largest absolute web area, which differed from both *M. menardi* (t = − 3.74, df = 6.57, *P* = 0.019) and *M. mengei* (t = − 3.86, df = 11.61, *P* = 0.0062), while the two closely related species had similar sized webs (t = − 0.62, df = 7.59, *P* = 0.816).

**Table 1:** Web characteristics of *Meta menardi*, *Metellina mengei* and *Tetragnatha montana.*

Web characteristics	*M. menardi*	*M. mengei*	*T. montana*	F^*a*^	*P*^*b*^
Sample size	19	29	25		
Absolute web area (cm^2^)	108 ± 11	132 ± 11	251 ± 25	9.73	0.007
Web asymmetry	− 0.02 ± 0.04	− 0.16 ± 0.02	− 0.17 ± 0.03	5.38	0.047
Web shape	0.05 ± 0.03	− 0.05 ± 0.02	− 0.06 ± 0.03	4.32	0.070
Average number of capture spiral turns	6.0 ± 0.4	15.0 ± 0.7	14.4 ± 0.6	30.70	< 0.001

Mean ± S. E. M.

^a^Statistical tests were conducted with a linear mixed effect model.

^b^*P* values were found with the Type II Wald F tests with Kenward-Roger degrees of freedom.

## Discussion

We observed, in accordance with our hypothesis, that webs of the cave-dwelling orb spider *Meta menardi* showed statistically discernible and visually apparent differences to the webs of related aboveground species. Most notable was the lack of the frame that usually enclose the capture spiral and radii of orb webs. This resulted in the radii acting as mooring threads by attaching directly to the surrounding rock substrates. Thus, these cave webs had a much larger number of mooring threads than standard orb webs, which could provide information to the spider about prey walking on the rock surface in vicinity of the web. To our knowledge, this study, utilizing the unique opportunities for quantifying foraging behaviour offered by spider webs, is one of very few studies on cave-dwelling animals that demonstrate a significant difference in behaviour compared to closely related surface-dwelling counterparts^6,38^.

Morphological adaptations to the subterranean environment have been studied in much greater details than behavioural adaptations with examples of convergent evolution in a number of traits including loss of eyes, loss of pigments and limb elongation^3,5,39^. To complement our behavioural studies, we therefore also compared limb elongation in the cave orb spiders given that they show no evidence of eye or pigment loss^15,40^. However, in our comparative study, *M. menardi*, contrary to our hypothesis, did not have the longest relative leg length for either leg I or III, although it did have statistically significantly longer relative leg lengths than its closest surface relative, *Metellina mengei*. The facultative cave spider *Metellina merianae* has relative leg lengths in between these two species^41^, so more detailed studies are needed to determine if some degree of limb elongation might be present in cave orb spiders. The large overall size of *M. menardi* could potentially also be viewed as an adaptation to the subterranean environment as an increased body size is a frequent morphological adaptation seen in cave arachnids^3^. *Meta* spiders are generally among the largest members of the family Tetragnathidae^15^, so their size could indicate some degree of morphological adaptation, although it is worth noting that large surface-dwelling araneid spiders are relatively common in the tropics.

In the Japanese cave orb spider, *Meta japonica*, a very similar web structure to the one we found for *M. menardi* was observed, showing an omission of a complete frame (number of frame sections, mean ± SD: 1.1 ± 0.6) and a similar number of radii which attached directly to the wall (14.6 ± 5.3)^31,42^. However, the number of capture spiral turns (17.0 ± 4.3) was considerably greater than recorded for *M. menardi* in this study (6.0 ± 1.7), suggesting that the mesh height would be lower. A similar number of radii (16.9 ± 3.0) and spiral turns (13.0 ± 3.0) were observed in the facultative cave spider *M. merianae*, but this species does not show any reduction of frame threads and only a few mooring threads connect the web to the cave wall^41^. Interestingly, the webs of *M. menardi* were more symmetrical than the webs of the two surface-dwelling species. However, as web asymmetry in tetragnathid spiders is closely linked to inclination with more vertically inclined webs being more asymmetric^43,44^, the symmetry of the cave webs is possibly due to these webs being more horizontally inclined (D. Simonsen, Pers. Obs.) than an adaptation to subterranean conditions per se. The webs of *M. menardi* were smaller than those of its surface relatives, although the webs of *M. mengei* and *M. menardi* were more similar to each other than either one was to *Tetragnatha montana.* Given that web area tends to increase with spider size^45,46^, our results are diametrically opposed to what was expected with the relative web area of *M. menardi* being significantly smaller than found in the other two species.

A relatively smaller web combined with the lower number of capture spirals and the slightly increased relative mesh height, suggests that the webs of *M. menardi* are not as important for prey capture as in other species. However, there was no difference in the number of radii, resulting in *M. menardi* having a higher density of radii and potentially more stopping pontential, which, combined with their relatively smaller capture area, may suggest that they rely on capturing fewer, but larger prey^47,48^. This is though not supported by the prey captured in their web, which seems to consist mainly of small gnats, mosquitoes and caddisflies^15,32^, whereas woodland *Tetragnatha* and *Metellina* are known to feed on relatively larger prey including large mosquitoes and tipulids^49,50^, although *Metellina mengei* from Wytham Woods seems to predominantly feed on small aphids^51^. Finally, aerial prey (mainly mosquitoes) has been observed to escape quickly from *M. menardi* webs^52^, suggesting they might not successfully retain larger and stronger insects should such prey be intercepted.

The diet of *M. menardi* has been confirmed, from several field studies, to consist of between 36 and 69% non-flying prey including slugs, spiders and millipedes^15,32,40,53^ compared to a diet predominantly consisting of flying prey observed in other orb spiders^18^. It is generally accepted that *Meta* spiders combine both traditional on-web prey capture with novel off web hunting to achieve their broadened diet^15,30,31,40^. However, a foraging behaviour involving hunting independent of the web seems unlikely as this would require evolving a completely new additional prey capture behaviour^15^. Instead, with the radii attaching to the wall and *M. menardi* residing in the hub of its web, as seems to be its usual position (D. Simonsen. Pers Obs)^52^, it would be able to detect the presence and direction of both prey flying into the web and walking prey colliding with the radii through the same sensory mechanism of vibrations transmitted through the radii^54,55^. This hypothesis is supported by the observation from Novak et al.^40^ that *M. menardi* “swiftly traced prey from its web onto the wall” after the prey had collided with the radii and by an unpublished pilot study by Peter Smithers (Pers. Comm. 2019) that indicated that *M. menardi* in the laboratory catch woodlice by running down and attaching silk threads to them after they walk into a radial thread. Thus, the observed lack of frame threads in the webs of cave orbs spiders from this study is likely to be a behavioural adaptation to broaden the diet in response to the rarity of flying prey in subterranean habitats. To confirm this hypothesis, either careful field work or laboratory experiments, which result in the direct observation of prey capture from the wall would be required combined with a comparative analysis of web structure and prey capture in one of the confirmed surface-dwelling *Meta* species; for example the endemic *M. stridulans* from the laurel forests of Madeira^56^.

The comparative approach adopted in this study was somewhat skewed as all cave spiders measured were late instar juveniles, while the aboveground spiders measured were all adults. However, while this may have impacted some of our quantitative results, it is unlikely to have had any influence on the qualitative differences and conclusions presented in this paper as orb webs generally do not undergo strong ontogenetic changes^57^. The changes they do show either relate to web asymmetry, as older and larger spiders build webs with larger lower parts due to faster gravity-assisted downwards running or to derivative web features such as free sectors not present in juvenile spiders^58–60^. The tetragnathids studied here, all built standard orb webs with the exception of the lack of frame threads in subadult cave spiders, and as they built inclined webs, the link between size and asymmetry is expected to be weak^43,44^. Thus, the major differences such as number of frames, number of radii and relative web size between the cave spider and the two aboveground spiders found in this study are therefore likely to be due to habitat rather than ontogeny, especially as observations on adult *Meta menardi* suggest they construct similar webs to those in our study^30,61^. However, we cannot rule out that the differences found in asymmetry are caused by ontogeny.

Orb spiders are known to show an impressive flexibility in their orb web designs in response to a large number of biotic and abiotic factors^27,35,62^, including in response to spatial constraints^63,64^ and to climatic variables^25^. Could the modification in web geometry in the current study therefore be explained by behavioural flexibility due to environmental differences rather than an evolved behavioural adaptation for foraging in caves? Would either of the terrestrial relatives produce similar webs if they found themselves in a subterranean habitat? The average temperature in the cave was 6 °C colder than in the wood and 10 °C colder than along the river. At colder temperatures, orb spiders build smaller webs with fewer capture spiral turns, but a similar number of radii^25^, which is similar to our findings. However, mesh height was also found to increase, contrary to our findings, and more importantly no changes in overall web structure are known to occur as a consequence of temperature. Similarly, wind, albeit not directly measured in this study, is likely to be lower in the caves than in the wood and along the river. Higher winds are known to lead to smaller webs with fewer capture spiral turns^25,65,66^, where we found the opposite with the smallest webs in the sheltered caves. Noteworthy is also that a field study on *M. mengei* did not find any differences in web design between webs built at the exposed woodland edge and webs built in the sheltered interior of the wood^67^. Again, wind is not known to affect the fundamental structure of the web. Yoshida and Shinkai^31^ suggested that the reduction of frame threads in *Meta japonica* is a result of building webs in small cavities on the cave wall and ceiling, as *M. menardi* is also observed to do. Behavioural flexibility seems to play some role as when the frame threads were present, they were predominantly found at larger distances from the rock wall (D. Simonsen, Pers. Obs.), potentially suggesting that the presence of a frame is dependent on the topology of the environment. However, orb spiders under spatial constraints in the laboratory attempt to maintain overall web area by elongating their webs to match the available space and by reducing mesh height, contrary to our findings for *M. menardi*^25,63,68^. In addition, most species facing spatial constraints do not change the structure of their webs, although the araneid *Eustala illicita* and the tetragnathid *Leucauge argyra* have been observed to attach radii directly to the surface when building orb webs in very constrained artificial frames and tubes^63,64^. These webs, however, only had small sections of frame threads missing, contrary to our findings of an almost complete elimination of frame threads. The anapid *Anapisona simoni* shows more dramatic changes in frame, radial and capture threads that are somewhat similar to the responses observed in this study^69^, but anapid webs are very different from tetragnathid webs as they are more three-dimensional and lacking the non-sticky scaffolding spiral. Overall, comparisons between strongly constrained spiders in the laboratory and cave spiders in the wild have only limited validity as natural caves typically offers a range of potential web sites as demonstrated by the normal cave orb webs of the sympatric *Metellina merianae*^41^. Finally, it is also possible that the observed changes occur as a direct response to the low food availability in the subterranean habitats. However, while orb spiders are known to alter life history and silk properties due to starvation^70,71^, only minor quantitative changes in orb geometry, including increased area, but reduced number of capture spirals, were found^72,73^ as opposed to the large-scale quantitative and qualitative differences observed in this study. In addition, *Meta menardi* does not show any specific physiological adaptations to starvation^74^. Overall, the changes we observed in the webs of *M. menardi* are unlikely to be caused by behavioural flexibility alone, although more detailed laboratory studies of web-building behaviour of the cave spider are needed to fully eliminate this possibility.

In conclusion, our findings that the cave orb spider showed significant departures in its web geometry compared to two related surface-dwelling orb spiders, especially in terms of a reduction of frame threads with radii attaching directly to the cave wall and ceiling, indicate that this behavioural modification could have evolved in response to the limited availability of flying prey and high relative abundance of walking prey in subterranean habitats. While more observations and experiments on the prey capture and web-building behaviour of cave orb spiders is needed, this is to our knowledge the first quantitative study to suggest novel behavioural adaptations in a terrestrial cave arthropod. This has broader implications since the cave spiders in our study only show limited evidence for morphological adaptations (no loss of eyes or pigment, but with some indication of minor limb elongation compared to their closest surface dwelling relative), which means these spiders could be in the initial stages of a full adaptation to the cave environment with behavioural adaptation preceding morphological adaptations (which might eventually evolve into a complete loss of the web and adaptations to off-web locomotion and prey capture). Thus, we suggest that *Meta* cave spiders could be a potential model organism for studying behavioural flexibility and the evolution of behaviour in energy-poor environments.

## Methods

### Study organisms

The European cave orb spider, *Meta menardi* (family Tetragnathidae), is a large spider with adult females measuring up to 15 mm in total length that inhabits the first 20–30 m of natural caves and artificial subterranean habitats including tunnels and mines, although it has also been found in cellars, tree crevices, under man hole covers and in cavities on scree slopes^15^. Since no species in the *Meta* genus, with the exception of the endemic *Meta stridulans* from Madeira, are known to occupy aboveground habitats, we compared the morphology and web geometry of *M. menardi* to two common sympatric aboveground tetragnathids; the closely related *Metellina mengei* (a fellow member of the Metainae subfamily) and *Tetragnatha montana* from the Tetragnathinae subfamily^15,33^. *Metellina mengei* is a small to medium-sized spider up to 6 mm in length that mainly inhabits humid woodland understory or shaded grassland, while *T. montana* is a medium to large-sized spider that can be up to 10 mm in total length and is found in humid woodland understory and in vegetation along streams and rivers.

### Sampling protocol

We sampled 19 M*. menardi* webs during 2 days in June 2018 at Creswell Crags, U.K. (53.264056 N, − 1.192111 W) from the twilight zone (here defined as 2–30 m from the entrance) of the following three limestone caves. Robin Hood cave is a large cave formed of several large channels and chambers extending to a depth of more than 50 m (53.262120 N, − 1.2009147 W). Church Hole cave is formed of a medium sized chamber at the entrance and a channel around 35 m long (53.262120 N, − 1.2009147 W). Pin Hole cave consists of a channel around 25 m long (53.261468 N, − 1.2025036 W). We sampled each accessible (i.e. not requiring climbing or speleological equipment) spider web encountered in the three caves. Inaccessible webs were usually built higher up on chamber walls or roofs and the microstructure of these sites did not differ noticeable from the accessible sites. The average (± SEM) temperature and humidity in the caves at the location of the sampled webs were measured with a Preciva LCD Digital Psychrometer as 13.4 °C ± 0.5 °C (N = 19) and 81.3% ± 1.8% R.H. (N = 19) respectively.

We sampled 29 M*. mengei* webs in Wytham Woods, U.K. (51.777889 N, − 1.337778 W) in May and June 2018. Webs sampled were located within 2 m either side of ten 50 m transects with a maximum of 10 webs from each transect. All transects were randomly chosen from ordnance survey maps before seeing the location of the transect. In order to minimize sampling bias, the transect was first walked without sampling following the protocol of Tew and Hesselberg^62^. On the second walk, we spent at least two minutes searching at different heights and angles around every two meters while sampling every orb web found. The average (± SEM) temperature and humidity in the woods at the location of the sampled webs were measured with a Preciva LCD Digital Psychrometer as 19.8 °C ± 0.5 °C (N = 29) and 74.4% ± 1.8% R.H. (N = 24) respectively.

We sampled 25 T*. montana* webs along the River Cherwell in Oxford University Parks, U. K. (51.763306 N, − 1.250222 W) between May and June 2018. Webs sampled were located within 2 m either side of five 50 m transects following the same protocol as when sampling *M. mengei* in Wytham Woods. The average (± SEM) temperature and humidity along the river at the location of the sampled webs were measured with a Preciva LCD Digital Psychrometer as 23.6 °C ± 0.5 °C (N = 25) and 58.6% ± 1.4% R.H. (N = 25) respectively.

For all locations, the resident spiders of the webs were collected and taken back to the laboratory, where they were stored in 70% ethanol before their ID was confirmed, morphological data collected and their life stage determined based on the absence or presence of the epigyne. While all *M. mengei* and *T. montana* collected were adult females, the collected *M. menardi* turned out to be mid to late instar juvenile females. In general, adult *Meta* spiders are encountered much less frequently than juveniles^75^.

### Data collection: size and leg length

The collected spiders of all three species were brought to the laboratory where the following measurements were taken using a digital Vernier calliper under a stereo microscope to a precision of 0.1 mm: total length of the spider, cephalothorax width, as a measure of spider size and the combined patella-tibia length of leg I and III.

### Data collection: web geometry

**Figure 5:**
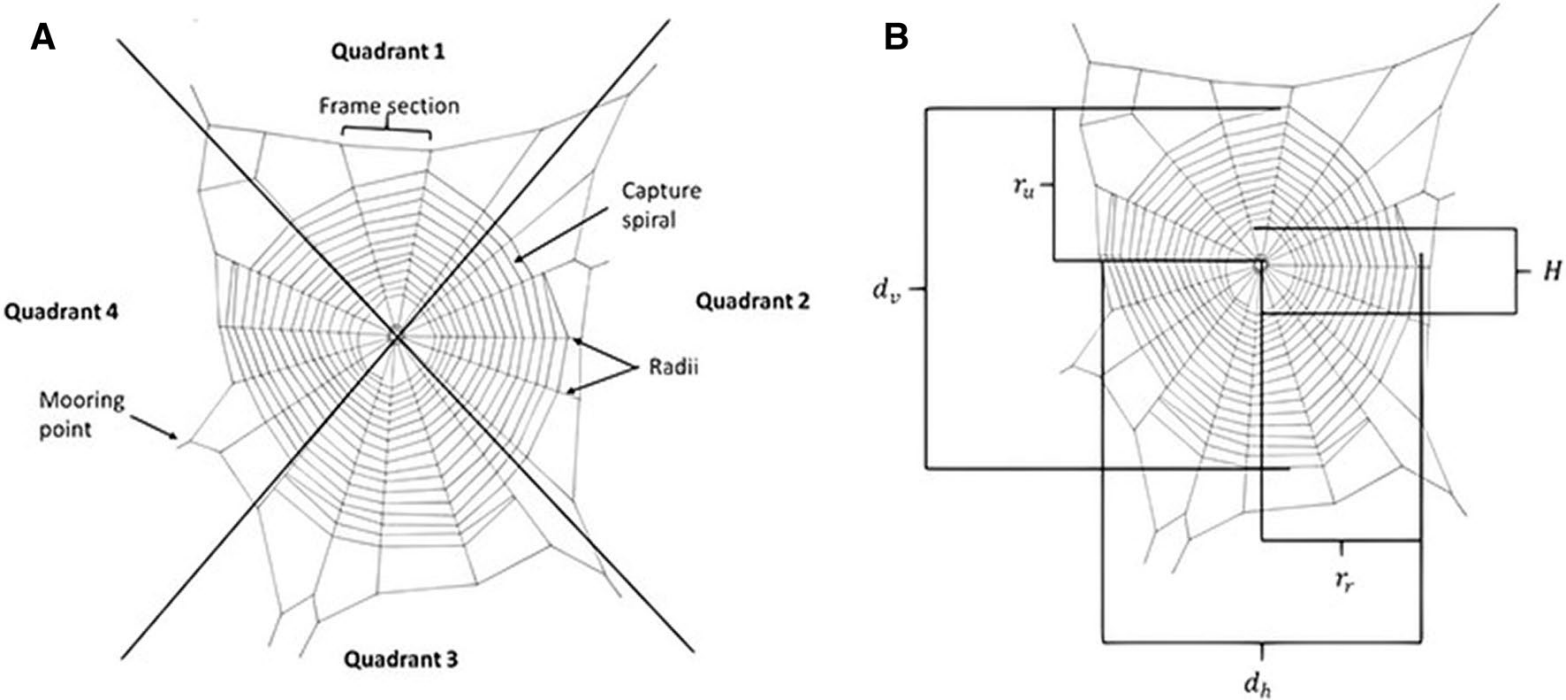
Schematic drawing of a typical tetragnathid orb web highlighting the variables measured in the study. (**A**) Shows the different silk thread types and how the web was divided into 4 quadrants. (**B**) Shows the measured variables: vertical (d_v_) and horizontal (d_h_) diameter, upper (r_u_) and lower radius (r_l_), and hub diameter (H).

All webs located in the field were misted with water to make the silk threads more visible and hence easier to measure. Five different length characters (Fig. 5A) were measured using a digital Vernier calliper to the nearest mm: (1) The vertical ($d_{v}$) and (2) horizontal diameters ($d_{h}$) were measured from the outermost and opposite spiral threads (3) The upper ($r_{u}$) and (4) horizontal radius ($r_{r}$) were measured from the centre of the central hub to the outermost spiral thread. Finally, (5) the vertical diameter of the central hub and free zone ($H$).

Count measurements (Fig. 5B) include the number of radii, the number of capture spiral turns along the mid radii in each of the 4 quadrants centres ($s_{Q1} ,s_{Q2} ,s_{Q3} ,s_{Q4}$), the number of frame threads and the number of mooring points (i.e. the number of mooring threads connecting the web to the surrounding substrate).

The measured web parameters allowed us to derive these further web characteristics:

Web area (the area of the capture spiral minus the free sector and hub) was estimated using the Ellipse–Hub formula^76^:

$$\left( {\frac{{d_{v} }}{2}} \right)\left( {\frac{{d_{h} }}{2}} \right)\pi - \left( \frac{H}{2} \right)^{2} \pi$$

The lower vertical radius ($r_{l}$) and left horizontal radius ($r_{le}$) were calculated as follows:

$$\begin{aligned} & d_{v} - r_{u} = r_{l} \\ & d_{h} - r_{r} = r_{le} \\ \end{aligned}$$

Web asymmetry was characterized by a value between − 1 to 1, with 0 indicating a perfectly vertical symmetric web and a negative value indicating that the hub is located closer to the top than the bottom of the web and was found with the following formula^58^:

$$\frac{{r_{u} - r_{l} }}{{r_{u} + r_{l} }}$$

Web shape was also characterized by a value between − 1 to 1, with 0 indicating a circular shape and a negative value indicating it is taller than it is wide and was found with this formula^77^:

$$\frac{{d_{h} - d_{v} }}{{d_{h} + d_{v} }}$$

Mean mesh height, which is the distance between two adjacent capture spiral turns, was measured using this formula, which we adapted from the formula in Herberstein and Tso^76^ in order to include the horizontal quadrants:

$$\frac{1}{4}\left[ {\frac{{\left( {r_{u} - \frac{H}{2}} \right)}}{{s_{Q1} - 1}} + \frac{{\left( {r_{l} - \frac{H}{2}} \right)}}{{s_{Q3} - 1}} + \frac{{\left( {r_{r} - \frac{H}{2}} \right)}}{{s_{Q2} - 1}} + \frac{{\left( {r_{le} - \frac{H}{2}} \right)}}{{s_{Q4} - 1}}} \right]$$

### Data analysis: morphology

To determine differences in morphology (total length, cephalothorax width, and patella-tibia length of leg I and leg III) between *M. menardi* and its surface-dwelling relatives, we performed a principal component analysis with the *prcomp()* function in R and used the *ggplot2* package^78^ to generate ordination plots. However, in order to investigate potential limb elongation in *M. menardi*, we followed Hesselberg and Simonsen^41^ to control for spider size by using relative leg length (patella-tibia lengths of leg I and III divided by cephalothorax width) as the response variables in linear mixed models where species was the fixed factor with location (transect number and cave ID) as a random factor. To ensure normality, the relative leg length of leg I in the comparison of all species was transformed with the natural logarithm. In an attempt to separate phylogeny and habitat, we found pairwise contrasts with Kenward-Roger degrees of freedom and the Tukey Method for *P* value adjustment with *emmeans()* function from the emmans package^79^ as a posthoc test, where the full model revealed significant differences between species. All models were built with the *lmer()* function from the lme4 package^80^ in R^81^. *P* values were found with the Type II Wald F-test.

### Data analysis: web geometry

Linear mixed models were developed following the approach described above for number of radii, absolute web area, number of mooring threads, web asymmetry, web shape, relative mean mesh height (mesh height divided by cephalothorax width) and relative web area (web area divided by cephalothorax width squared) with species as a fixed factor and location as a random factor. To ensure normality, the relative web area was logarithmic transformed. We used the same posthoc test described above for pairwise species comparisons, where the full model showed species was a significant variable. All models were built with the *lmer()* function from the lme4 package^80^ in R^81^. *P* values were found with the Type II Wald F-test.
